# Low serum IgE is associated with an increased risk of chronic lymphocytic leukemia: a large retrospective cohort study

**DOI:** 10.3389/fimmu.2026.1772840

**Published:** 2026-03-11

**Authors:** Ramon Cohen, Daniel Elbirt, Shay Nemet, Alena Kirzhner, Tal Schiller, Haitham Abu Khadija, Shira Bezalel-Rosenberg, Ilan Asher, Keren Mahlab-Guri, Ofir Wolach, Liron Hofstetter

**Affiliations:** 1 Department of Internal Medicine B, Kaplan Medical Center, Faculty of Medicine, Hebrew University of Jerusalem, Jerusalem, Israel; 2 Department of Clinical Immunology, Allergy and AIDS, Kaplan Medical Center, Hebrew University of Jerusalem, Jerusalem, Israel; 3 Department of Internal Medicine A, Kaplan Medical Center, Faculty of Medicine, Hebrew University of Jerusalem, Jerusalem, Israel; 4 Institute of Endocrinology, Diabetes and Metabolic disease, Wolfson Medical Center, Gray Faculty of Medical and Health Sciences, Tel Aviv University, Tel Aviv, Israel; 5 Department of Cardiology, Kaplan Medical Center and Faculty of Medicine, Hebrew University of Jerusalem, Jerusalem, Israel; 6 Institute of Hematology, Davidoff Cancer Centre, Rabin Medical Centre, Petah Tikva, Gray Faculty of Medical and Health Sciences, Tel Aviv University, Tel Aviv, Israel

**Keywords:** atopy, chronic lymphocytic leukemia, CLL, IgE, leukemia

## Abstract

**Introduction:**

Chronic lymphocytic leukemia (CLL) is the most prevalent adult leukemia in the western world. Its pathophysiology is intertwined with immune dysfunction. Emerging evidence suggests an inverse association between serum immunoglobulin E (IgE) and hematologic malignancies, but previous studies linking low IgE to CLL risk were limited by small cohorts and a lack of adjustment for confounding factors, particularly hypogammaglobulinemia.

**Purpose:**

This study aimed to evaluate the association between low serum IgE levels and the future development of CLL in a large, real-world cohort, while accounting for other immunoglobulins and confounding factors.

**Methods:**

We conducted a retrospective quantitative observational study of 118,740 adults from a large health maintenance organization. The primary exposure was a baseline IgE level of less than 25 IU/mL. We used Kaplan-Meier curves and a multivariable Cox proportional hazards model to assess the association between low IgE and CLL diagnosis over a seven-year follow-up period, adjusting for age, sex, and other potential confounders, including hypogammaglobulinemia and atopy-related conditions.

**Results:**

A serum IgE level of less than 25 IU/mL was significantly associated with an increased hazard of developing CLL (HR = 1.94, 97.5% CI: 1.47–2.56). This association persisted after adjusting for all confounding variables. Established risk factors, such as older age (HR = 1.07) and male sex (HR = 1.82), were also significant. Kaplan-Meier curves showed a sustained and a statistically significant increased risk in the low IgE group throughout the follow-up period.

**Conclusion:**

Lower serum IgE levels are independently associated with an increased risk of developing CLL.

## 1 Introduction

Chronic lymphocytic leukemia (CLL) is the most common leukemia in adults in Western countries and represents a clinically heterogeneous disease with variable outcomes. Like others hemato-oncologic disease, the pathophysiology of CLL is deeply intertwined with immune dysfunction and dysregulation (1–3), making immunological markers increasingly relevant in the early identification and prognosis of the disease (4). Among these markers, immunoglobulin E (IgE), traditionally associated with allergic responses, is gaining attention for its potential role in hematologic malignancies (5, 6).

Emerging studies have proposed an inverse relationship between total serum IgE levels and the development of hematologic cancers, including CLL (7). An analysis based on the European Prospective Investigation into Cancer and Nutrition (EPIC) cohort (8) found that individuals with lower prediagnostic IgE levels were more likely to develop CLL, with an association observed for levels below 20 IU/mL (OR = 0.49 for normal IgE and 0.13 for high IgE vs. low IgE) (8). These findings support the hypothesis that low IgE may be a preclinical marker of CLL risk. However, data specifically linking IgE to CLL risk are sparse and often limited by small cohorts, or a lack of adjustment for immunoglobulin deficiencies that may precede CLL (8–10). A study considering hypogammaglobulinemia and the risk of cancer showed a higher risk for patients with IgE deficiency (<2.5 IU/mL) but was not specific to CLL (11, 12). Only some studies considered other potential risk factors, such as atopy, smoking, alcohol, or obesity, that can also influence IgE levels (13–16).

This research aims to clarify the association between lower IgE levels and the future development of CLL, while explicitly accounting for other immunoglobulins and confounding factors in a large sample. By evaluating IgE levels in relation to CLL risk in a well-characterized cohort, this study seeks to determine if lower IgE may be predictive for early risk assessment.

## 2 Materials and methods

### 2.1 Research design

To identify a clinically relevant IgE threshold and the timeframe, we based our decision on prior literature (8), we pre-specified <20 IU/mL for a seven years follow-up as the primary threshold for low IgE. We then check higher threshold. To ensure robustness, the IgE thresholds higher than 20 IU/mL had to remain statistically significant and persist for at least seven years in both genders using Kaplan-Meier curves. Except for age, all variables were categorical. For categorical covariates, patients with missing data were grouped with those in the ‘not present’ category. To ensure data quality, all patients born in another country or who quit CHS were excluded. To assess our assumptions, we used piecewise Cox proportional hazards and calculated the risk ratio at 3 and 7 years to ensure stability. Patients with HIV or who received drugs that can influence IgE levels were also excluded. Follow-up to 10 years was also checked. Considering the link between allergy, IgE, and CLL, we performed a sub-analysis of atopic and non-atopic groups using Kaplan-Meier curves (more information is in the supplement).

This study was conducted in accordance with ethical standards and received approval from the relevant institutional review board. All patient data were de-identified prior to analysis. As this was a retrospective analysis of routinely collected data, informed consent was waived in accordance with local regulations.

### 2.2 Patient population

Patient data were derived from Clalit Health Services (CHS), a large health maintenance organization with more than 4.8 million patients and 1500 clinics. Data extraction and statistical analyses were conducted using Wiser software (version Alfa, Rehovot) (17–19). Data collection included patients for whom the blood samples with IgE measurements were available (a diagram of eligibility is provided in the supplement). The first IgE measurement was used as index date (cox regression with all laboratories – even several for the same patient is found in the supplement). Because of the large population-based cohort, *a priori* power calculations were not undertaken. Rather, we maximized statistical power and external validity by including all available eligible patients in the database. Data extraction was performed on March 4, 2025. Characteristics of the populations are found in **Table 1A**.

**Table 1A:** Characteristics of patients with IgE lower and higher than 25 IU/mL and the population for the cox regression (categorial).

Feature	≤ 25 IU/mL (n = 36,429)	>25 IU/mL (n = 82,311)	P-value*	Cox group (n = 118,740)
Sex, male	9,814 (26.94%)	33,373 (40.55%)	<0.01	43,187 (36.37%)
Atopic dermatitis	576 (1.58%)	1,579 (1.92%)	<0.01	2,155 (1.81%)
Asthma	3,655 (10.03%)	13,637 (16.57%)	<0.01	17,292 (14.56%)
Allergic rhinitis	6,216 (17.06%)	17,248 (20.95%)	<0.01	23,464 (19.76%)
Smoking	3,942 (10.82%)	11,857 (14.41%)	<0.01	15,799 (13.31%)
Obesity	4,945 (13.57%)	11,303 (13.73%)	0.46	16,248 (13.68%)
Urticaria	1,483 (4.07%)	4,908 (5.96%)	<0.01	6,391 (5.38%)
Hypogammaglobulinemia	370 (1.02%)	436 (0.53%)	<0.01	806 (0.68%)
Alcoholic cirrhosis	152 (0.42%)	310 (0.38%)	0.30	462 (0.39%)
IgA deficiency	222 (0.61%)	138 (0.17%)	<0.01	360 (0.30%)
IgM deficiency	595 (1.63%)	1,241 (1.51%)	0.11	1,836 (1.55%)

*Chi-square.

### 2.3 Study objective

This study aimed to evaluate the association between lower level IgE levels and the development of CLL among adults aged 40 to 80 years, diagnosed between January 1, 2004, and December 31, 2024. Participants were followed for up to seven years from the index date. Sub-analysis about atopy and follow-up for 10 years are found in the supplement.

### 2.4 Outcomes and follow-up

The primary outcome was time to CLL diagnosis within the seven-year follow-up period. Patients were observed from their index date until the earliest occurrence of one of the following: a confirmed CLL diagnosis, loss to follow-up, or the end of the study period. Demographic variables (age and sex), prior diagnoses, and laboratory data were analyzed. Exposure variables were categorized based on their timing relative to the index date.

### 2.5 CLL case definition

The CLL cohort included patients identified with the ICD-9 diagnostic code 204.1 (chronic lymphocytic leukemia). To ensure data accuracy, lymphocyte counts (both absolute and relative percentages) were compared with values from the general population. Elevated lymphocyte levels were required in proximity to the CLL diagnosis (see **Supplementary Materials** for detailed diagnostic criteria).

### 2.6 Confounders and risk factors

Confounders and risk factors were considered from birth to the index date, except for urticaria, which was considered for up to 3 months before the index date (more information is in the supplement).

### 2.7 Laboratory data

Laboratory Data of Immunoglobulin levels were tested within 3 months of the blood sample with IgE. This timeframe was selected to better capture the clinical context in which laboratory abnormalities may first become apparent. Hypogammaglobulinemia was defined as serum IgG <700 mg/dL. IgM deficiency was defined as serum IgM <40 mg/dL, and IgA deficiency was defined as a level less than 70 mg/dL. These thresholds were selected based on reference laboratory ranges because they are typically used to define whether a patient has hypoglobulinemia (IgG, IgA, IgM).

### 2.8 Assays used for serum IgE testing in retrospective analysis

At CHS, the methodology for serum IgE testing varied over the study period. Prior to 2007, CHS operated without a central laboratory, and IgE testing was outsourced to multiple small laboratories. As none of these laboratories are currently operational, details regarding the specific assays employed during that time are unavailable (cox regression from 2007 to 2023 in provided in the supplement). From July 1, 2007, to July 15, 2019, testing was performed using the ADVIA Centaur Immunoassay System. Since July 15, 2019, the Immulite system has been utilized (20).

### 2.9 Statistical analysis

#### 2.9.1 Kaplan-Meier curve

**Figure 1:**
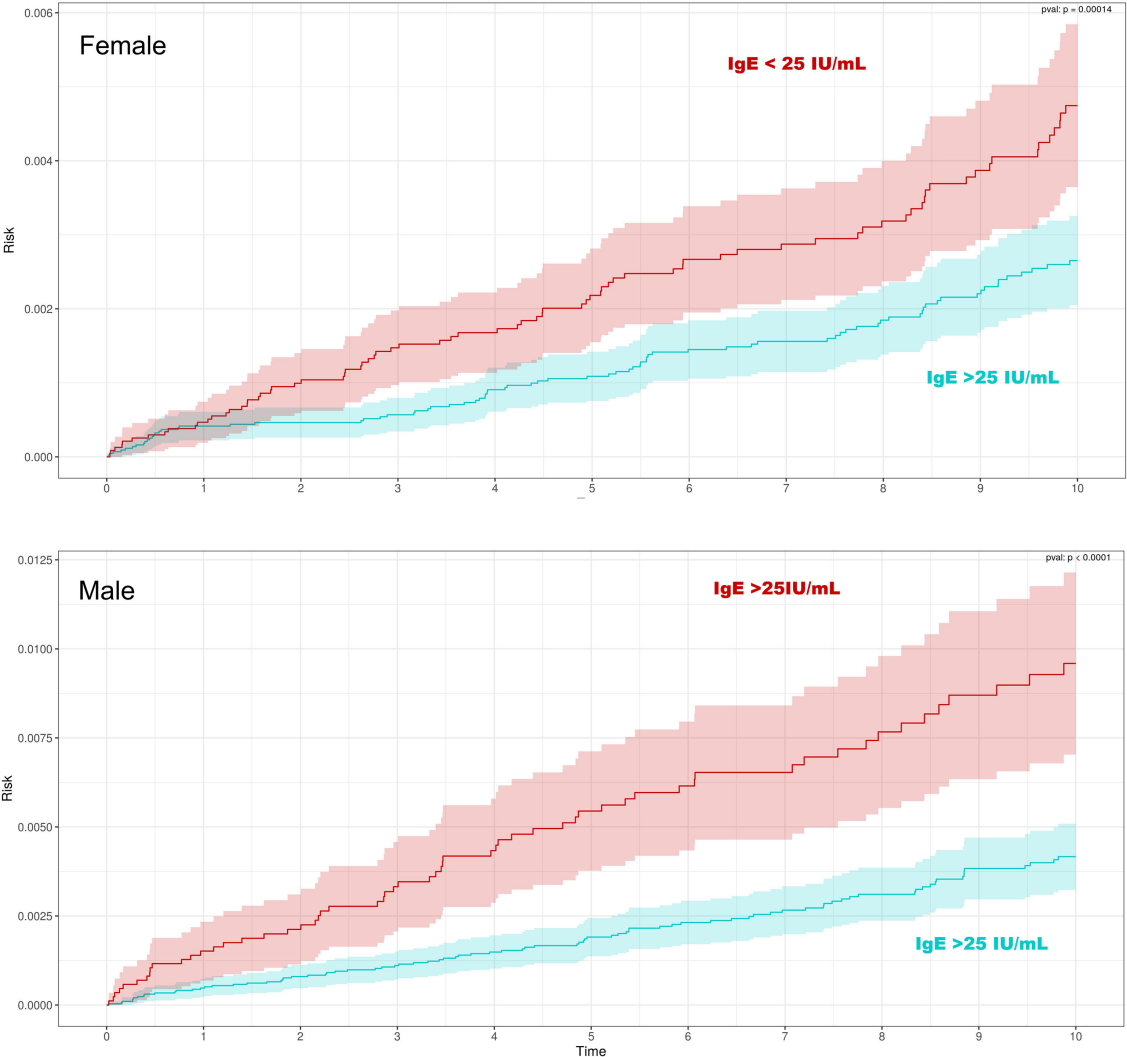
Cumulative risk of chronic lymphocytic leukemia (CLL) according to baseline serum IgE levels, stratified by sex. Kaplan–Meier curves show the 7-year cumulative risk of CLL in females (top panel) and males (bottom panel), comparing individuals. The Kaplan-Meier curves are statistically significant (p-value less than 0.01) for both sexes.

A Kaplan-Meier curve was generated for the ≤ 25 IU/mL group and the >25 IU/mL group. Separate curves were generated for males and females, and confidence intervals (95% CI) were plotted (see **Figure 1**). The follow-up period was 7 years. The log-rank test was used to compare survival distributions between groups. Group characteristics (**Table 1A**) were also compared using chi-square tests. Statistical significance was set at p < 0.05 (calculate with Chi-square).

#### 2.9.2 Cox proportional hazards model

A multivariate Cox regression model was built using the first blood sample with IgE taken when patients were aged 40 to 80 years (**Table 1B**). The outcome variable was the time to the development of CLL during a seven-year period. The primary exposure variable was the presence of IgE levels less than 25 IU/mL. Obesity, smoking, alcoholic cirrhosis, asthma, atopic dermatitis, allergic rhinitis, hypogammaglobulinemia, IgM deficiency, IgA deficiency, gender, and age were included as covariates. Censoring was applied for patients who were lost to follow-up or who did not develop CLL within the seven-year study period. Adjusted hazard ratios (HR) with 97.5% confidence intervals (CI) were reported. Statistical significance was defined as an HR that did not include the value 1. The Cox regression with IgE levels less than 20 IU/mL is in the **Supplementary Materials**.

**Table 1B:** Median of continuous variables of patients with IgE lower and higher than 25 IU/mL and the population for the cox regression.

Variable	≤ 25 IU/mL	>25 IU/mL	Cox group
Age, years	58.31	57.57	57.80
IgM, mg/dL	95	94	95
IgG, mg/dL	1,117	1,182	1,162
IgE, IU/mL	11.30	110.00	57.00
IgA, mg/dL	213	245	234

#### 2.9.3 Risk ratio at years 3 and 7

Risk ratios were calculated to compare cumulative event rates between the higher and lower exposure groups at years 3 and 7 of follow-up. The cumulative incidence for each group was determined by dividing the number of patients who experienced events by the total number of patients at risk at baseline (sum of patients with cumulative events and patients still under observation at each time point).

We assumed that environmental factors like genetic factors, infections, prenatal factors, environmental factors (21–23) were mitigated due to the large number of patients included in the study.

## 3 Result

In total, 118,740 individuals were included in the Cox regression analysis. Among them, 36,429 had serum IgE levels <25 IU/mL, and 82,311 had IgE levels ≥25 IU/mL. For the Kaplan–Meier analyses (follow-up up to 7 years), 224 cases of chronic lymphocytic leukemia (CLL) were identified. Of these, 119 cases occurred in the higher-IgE group (82,311 individuals) and 105 cases in the lower-IgE group (36,429 individuals). This distribution reflects both the underlying cohort size and the event rates across IgE strata, forming the basis for the comparative risk estimates reported.

The highest threshold of IgE that remained statistically significant for the development of CLL using the Kaplan–Meier analysis was a level of 25 IU/mL (for 7 years).

### 3.1 Kaplan Meier curve

**Figure 1**: Kaplan-Meir curves.

### 3.2 Cox regression

Age and male sex were associated with increased risk, with hazard ratios (HRs) of 1.07 and 1.82, respectively. Hypogammaglobulinemia present at the index date was strongly associated with subsequent CLL, with an HR of 4.09 (95% CI 1.93–8.65); however, this association was no longer observed when hypogammaglobulinemia was assessed more than two years before CLL diagnosis. In contrast, low IgE levels remained significantly associated with CLL risk both when the index date was defined from 0–7 years before diagnosis and when restricted to 2–7 years before diagnosis. Alcoholic cirrhosis of liver was excluded from the cox regression considering the low number of patients with CLL (4 patients) and the unstable HR (0; 0- inf). In the supplement can be found the complete cox regression with level IgE based on the literature: Low IgE (<20 IU/mL): 2.19 (1.65–2.89) (**Table 2**).

**Table 2:** Cox regression analysis of 7 and 10 year incidence of CLL according to baseline IgE levels.

Feature	HR (97.5% CI) 0 to 7 years	HR (97.5% CI) 2 to 7 years	0–10 years HR (97.5% CI)
**Age (per year)**	**1.07 (1.05–1.08)**	**1.06 (1.04–1.08)**	**1.06 (1.05–1.07)**
**Sex, male**	**1.82 (1.38–2.40)**	**1.88 (1.32–2.69)**	**1.66 (1.31–2.09)**
Atopic dermatitis	0.50 (0.12–2.02)	NE	0.67 (0.25–1.81)
Asthma	0.88 (0.58–1.32)	1.16 (0.72–1.88)	0.85 (0.59–1.20)
Allergic rhinitis	1.13 (0.80–1.59)	1.29 (0.85–1.96)	0.98 (0.72–1.32)
Smoking	1.11 (0.75–1.62)	1.12 (0.68–1.84)	1.30 (0.94–1.78)
Obesity	1.01 (0.68–1.49)	0.84 (0.49–1.44)	0.77 (0.19–3.11)
Urticaria	0.90 (0.46–1.77)	1.18 (0.55–2.54)	0.80 (0.45–1.43)
IgM deficiency	1.57 (0.77–3.21)	1.19 (0.37–3.86)	1.52 (0.82–2.83)
IgA deficiency	2.22 (0.74–6.65)	2.01 (0.26–15.56)	**3.56 (1.52–8.36)**
**Hypogammaglobulinemia**	**4.09 (1.93–8.65)**	0.78 (0.10–5.99)	**2.39 (1.13–5.08)**
**IgE lower than 25 IU/mL**	**1.94 (1.47–2.56)**	**1.83 (1.28–2.62)**	**1.93 (1.53–2.44)**

NE, Non estimable. Bold values represent statistically significant results.

### 3.3 Risk ratio at years 3 and 7

At 3 years of follow-up, the risk ratio of development CLL was 0.398, indicating a 60.2% relative risk reduction in the >25 UI/mL group compared to the ≤ 25 IU/mL group (cumulative incidence: 0.089% vs. 0.223%, respectively). By 7 years, the risk ratio increased to 0.493, corresponding to a 50.7% relative risk reduction (cumulative incidence: 0.278% vs. 0.564%, respectively). The attenuation of the protective effect over time suggests that the benefit associated with higher exposure may diminish with extended follow-up, although the higher exposure group maintained significantly lower event rates throughout the observation period. These findings demonstrate a sustained, albeit decreasing, protective association that persists over long-term follow-up, with the most pronounced benefit observed in the early years after exposure initiation.

## 4 Discussion

This large retrospective cohort study provides supporting evidence regarding the role of serum IgE in the development of CLL. The principal finding is that lower serum IgE levels (<25 IU/mL) are associated with a markedly higher hazard of developing CLL over a seven-year period (HR = 1.94; 97.5% CI: 1.47–2.56). This association still be statistically significant after 10 years. Established risk factors such as older age (HR 1.07) and male sex (HR 1.82) were reaffirmed (24).

Our primary finding of an inverse association between lower IgE levels and CLL risk strongly aligns with and expands upon key studies in the emerging field of “AllergoOncology”. It also aligns with the nested case–control analysis from the European Prospective Investigation into Cancer and Nutrition (EPIC) cohort (25). However, our study provides further advancement by demonstrating that this protective effect persists after adjusting for other immunoglobulin isotypes (IgG, IgA, IgM), confounding factors, and atopy-related comorbidities, thereby isolating IgE’s independent role. The association for development of CLL remains significant after 7 years and when IgE is used as a continuous variable.

Several mechanisms regarding the role of IgE in the development of cancer, have been proposed. Preclinical and translational studies demonstrate that IgE can enhance anti-tumor immunity by engaging FcϵRI-expressing effector cells (such as monocytes, macrophages, and dendritic cells) to mediate antibody-dependent cellular cytotoxicity, phagocytosis, and pro-inflammatory responses within the tumor microenvironment. IgE-based monoclonal antibodies targeting tumor antigens have shown superior recruitment and activation of macrophages compared with IgG, leading to tumor cell killing and favorable modulation of the immune milieu (26–28). Additionally, IgE/FcϵRI-mediated antigen cross-presentation by dendritic cells can prime cytotoxic T lymphocytes, further enhancing anti-tumor responses (29).

The association between hypogammaglobulinemia and CLL risk in our adjusted model is consistent with previous reports suggesting that hypogammaglobulinemia represents a later event in CLL pathogenesis and is a well-recognized complication of CLL, rather than a predisposing risk factor. Prospective data demonstrate that hypogammaglobulinemia is rarely present before a CLL diagnosis and typically emerges closer to or after the onset of clinically detectable disease, with most cases occurring within three years prior to diagnosis or later (30). Indeed, in the sub-analysis, the hypogammaglobulinemia association lost its statistical significance when we measured Cox regression from2 to 7 years (see supplement). The mechanisms leading to hypogammaglobulinemia in CLL include impaired immunoglobulin production due to dysfunctional B-cell receptor signaling, immune dysregulation, and the influence of suppressive cytokines in the tumor microenvironment (31, 32).

Our study also shows that atopy tends to protect against CLL. The inverse association between atopy and the risk of CLL is known and likely due to the tendency of patients with atopy to have higher levels of immunoglobulin IgE (12, 33, 34). In our sub-analysis of the atopic and non-atopic groups, we also found that patients with lower IgE levels were at a higher risk for CLL (see supplement).

This study offers several contributions. First, it provides robust epidemiological evidence from a large, real-world cohort for the association of lower IgE levels with CLL development, strengthening the association by comprehensively adjusting for critical confounding factors. Second, our findings support the emerging concept of ‘immunological fitness,’ where the integrity of specific immune pathways, rather than the mere absence of a general deficiency, is a key determinant of susceptibility to hematological malignancies. Third, the study contributes tangible evidence to the field of AllergoOncology, demonstrating that immune mediated processes traditionally associated with allergy may have protective effects against certain cancers. From a practice perspective, by refining a potential IgE threshold and highlighting its independent predictive value, these findings could inform the future development of more personalized risk assessment strategies and clinical guidelines for CLL screening.

Several limitations must be acknowledged. First, our retrospective cohort is restricted to patients who underwent IgE testing in clinical practice. Importantly, CLL itself does not represent a clinical indication for IgE testing, reducing the likelihood of reverse causation. The design precludes causal inference and may be subject to residual confounding from unmeasured variables. Second, selection bias may be present because IgE measurements were clinically indicated rather than systematically screened, potentially enriching the cohort for patients with allergic or immunological conditions and limiting generalizability. Third, although our primary threshold (<20 IU/mL) was derived from prior literature, we explored nearby thresholds to assess robustness. This data-driven element introduces potential bias, but the consistency across alternative thresholds and continuous modeling supports the validity of the observed association. The use of the 25 IU/mL threshold, while based on statistical analysis, may not represent the optimal clinical threshold and requires external validation. Monoclonal B-Cell lymphocytosis measurement was not available. Nevertheless, the large sample size and the consistency of our findings with existing literature provide reassurance about their validity.

The observed association between low serum IgE levels and an increased risk of CLL highlights several avenues for future investigation. First, identifying which patients might benefit from routine IgE screening or longitudinal follow up remains a critical step. Further studies are needed to determine if 25 IU/mL is clinically relevant. In addition, it is essential to distinguish between individuals with persistently low IgE levels due to a primary or immunologic profile and those with a secondary decline later in life. These two subgroups may have different biological underpinnings and prognostic implications. Finally, the possibility of a shared genetic or epigenetic pathway linking reduced IgE production and CLL susceptibility warrants exploration. Genome-wide association studies and immunophenotypic profiling may help elucidate common mechanisms and refine early detection strategies in at-risk populations.

In summary, this large-scale retrospective cohort study provides robust evidence of an association between lower serum IgE levels and the future development of CLL in adults between 40 and 80 years of age. While the mechanisms underlying this protective effect require further investigation, these results support the emerging paradigm of AllergoOncology and suggest that IgE measurement could serve as a valuable biomarker in personalized CLL risk assessment. This work contributes to our understanding of the complex relationship between immune function and hematological malignancy, These findings highlight the need for future prospective studies with larger sample sizes and broader IgE stratification to better delineate threshold effects and inform screening strategies. It also supports the need for further study on the role of IgE in oncology, for example, to explore a potential causal link between IgE deficiency and CLL.

## Data Availability

The original contributions presented in the study are included in the article/**Supplementary Material**. Further inquiries can be directed to the corresponding author.
