# Supplementary material for: Low serum IgE is associated with an increased risk of chronic lymphocytic leukemia: a large retrospective cohort study
Source: Front Immunol. 2026 Mar 11;17:1772840. doi: 10.3389/fimmu.2026.1772840 (PMC13013483; doi:10.3389/fimmu.2026.1772840)
Supplement: Supplementary file 1 [file DataSheet1.pdf]

Supplementary Material

PART A: Additional Information About the Study

We selected Kaplan-Meier curves and Cox proportional hazards modeling as complementary methods for survival analysis. Kaplan-Meier analysis provides a visual and non-parametric description of time-to-event data, allowing for a straightforward comparison of survival probabilities between groups without assuming a particular distribution. It enables the early identification of trends and potential violations of the proportional hazard's assumption. In contrast, Cox regression offers the ability to adjust for multiple confounders simultaneously, providing an estimate of the independent effect of the exposure variable on the hazard of CLL. It also yields quantitative effect sizes (HRs) and supports statistical inference by combining both methods, we obtain a comprehensive visual and statistical assessment of the relationship between exposure and outcome—unadjusted and adjusted—thereby ensuring robust and clinically interpretable results.

To identify a clinically relevant IgE threshold, based on prior literature, we pre-specified <20 IU/mL as the primary threshold for low IgE. To evaluate robustness, we also examined adjacent higher thresholds and modeled IgE continuously. These complementary approaches allowed an assessment of whether the association between low IgE and CLL was consistent across different analytic strategies. To ensure robustness, the IgE thresholds higher than 20 IU/mL had to remain statistically significant and persist for at least seven years in both genders in the Kaplan-Meier curve. This criterion was chosen to prioritize early discrimination and facilitate earlier risk stratification, which allows for more efficient patient follow-up by focusing on individuals with persistently low IgE levels. The identified threshold was then applied in a multivariable Cox proportional hazards regression, thereby confirming the association in an independent subsample while limiting overfitting for the higher IgE threshold. Except for age, all variables were categorical. For categorical covariates, patients with missing data were grouped with those in the ‘not present’ category. This approach ensured that all patients were retained in the Cox regression analyses, while acknowledging that missing values were not modeled separately (complete case analysis is also in the supplement). To

ensure data quality, all patients born in another country or who quit CHS were excluded. To assess the assumptions of the Cox regression, we used piecewise Cox proportional hazards and calculated the risk ratio at 3 and 7 years to ensure stability. In addition, we performed a Cox regression restricted to the period from 2 to 7 years after the index date to minimize the risk of reverse causation and outcome-driven bias. We performed a cox regression with patients from 2007 to 2023, considering the lack of uniformity in the laboratory before 2007. Patients with HIV or who received drugs that can influence IgE levels were also excluded. Considering the link between allergy, IgE, and CLL, we performed a sub-analysis of atopic and non-atopic groups using Kaplan-Meier curves. To enhance diagnostic validity, absolute lymphocyte counts and relative lymphocyte percentages were cross-checked against population reference values. Confirmation of elevated lymphocyte levels in temporal proximity to the recorded CLL diagnosis was required (more information is also in the supplement).

As this was a retrospective analysis of all eligible adults within the Clalit Health Services database who had serum IgE testing between 2005 and 2023. Instead, we leveraged the full available cohort, which provides substantial statistical power due to its large size and comprehensive follow-up. The precision of estimates is therefore reflected in the reported confidence intervals rather than in formal power calculations.

Risk Ratio at years 3 and 7

Risk ratios were calculated to compare cumulative event rates between the higher and lower exposure groups at years 3 and 7 of follow-up. The cumulative incidence for each group was determined by dividing the number of patients who experienced events by the total number of patients at risk at baseline (sum of patients with cumulative events and patients still under observation at each time point). Risk ratios were computed as the ratio of cumulative incidence in the higher exposure group to that in the lower exposure group, with values less than 1.0 indicating reduced risk in the higher exposure group. Patients who remained event-free and continued follow-up at each time point were identified from Kaplan-Meier survival analysis, ensuring accurate denominator calculations for risk estimation.

1. **Diagram of eligibility for cox regression and Kaplan-Meier curves**

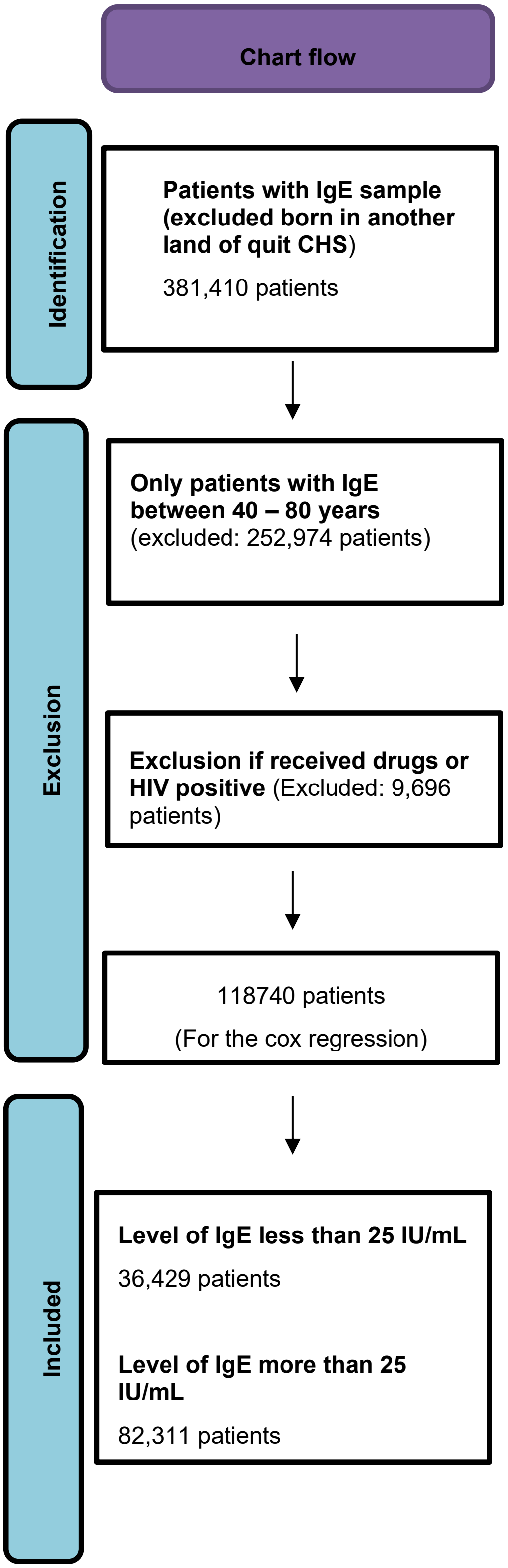

<u>Number of cases of CLL</u>

Cox regression group: 224 patients

Group with IgE higher than 25 IU/mL: 119 patients (in 82,311 patients)

Group with IgE lower than 25 IU/mL: 105 patients (in 36,429 patients)

2. Comparison of Lymphocyte Counts and Percentages Between Cases (CLL patients) and Controls (around diagnosis)

Feature	CLL, Mean (SD)	Controls, Mean (SD)	CLL, Median	Controls, Median
**Absolute lymphocyte count ($\times 10^9$/L)**	15.49 ± 23.56	2.19 ±1.63	8.80	2.06
**Lymphocyte percentage (%)**	57.20 ±19.50	30.76 ±8.58	60.40	30.50
**Atypical lymphocytes (Abs, $\times 10^9$/L)**	1.93 ± 6.33	0.28 ± 0.41	0.60	0.20
**Atypical lymphocytes (%)**	6.09 ± 8.44	3.64 ± 4.12	3.00	2.00

3. **Comparison of CBC and chemistry between Cases (CLL patients) and Controls (around diagnosis)**

Feature	CLL, Mean (SD)	Controls, Mean (SD)	CLL, Median	Controls, Median
**IgE (IU/mL)**	207.91 ± 542.81	428.03 ± 786.64	30.50	63.40
**IgM (mg/dL)**	90.48 ± 225.49	119.35 ± 102.75	51.20	86.00
**IgG (mg/dL)**	1008.42 ± 453.74	1223.31 ± 350.50	950.00	1188.00
**IgA (mg/dL)**	170.10 ± 175.15	261.06 ± 133.68	143.00	244.00
**Globulin (g/dL)**	2.65 ± 0.56	2.98 ± 0.44	2.60	2.96
**Albumin (g/dL)**	4.19 ± 0.46	4.21 ± 0.36	4.24	4.23
**Basophils (Abs, ×10⁹/L)**	0.41 ± 3.08	0.03 ± 0.04	0.10	0.02
**Eosinophils (Abs, ×10⁹/L)**	0.22 ± 0.24	0.21 ± 0.16	0.20	0.20
**Platelets (×10⁹/L)**	208.00 ± 86.45	248.26 ± 72.38	198.00	241.00
**WBC (×10⁹/L)**	26.48 ± 33.01	7.27 ± 2.40	17.05	6.90
**Hemoglobin (g/dL)**	12.88 ± 1.89	13.52 ± 1.56	13.10	13.50

4. Comparison of CBC and chemistry at index date (groups less and more than 25 IU/L)

Feature	IgE ≤ 25 IU/mL Mean ± SD	IgE > 25 IU/mL Mean ± SD	IgE ≤ 25 IU/mL Median	IgE > 25 IU/mL Median
**Atypical lymphocytes (Abs, ×10⁹/L)**	0.46 ± 0.82	0.33 ± 0.54	0.20	0.16
**IgE (IU/mL)**	**12.06 ± 7.14**	**322.22 ± 1178.97**	**11.30**	**110.00**
**Atypical lymphocytes (%)**	5.15 ± 7.00	4.06 ± 4.70	3.00	2.00
**IgM (mg/dL)**	120.80 ± 127.24	116.49 ± 107.36	96.00	94.00
**IgG (mg/dL)**	1169.23 ± 402.57	1231.54 ± 372.30	1115.00	1181.00
**Globulin (g/dL)**	2.91 ± 0.47	3.01 ± 0.46	2.90	3.00
**Albumin (g/dL)**	4.22 ± 0.35	4.18 ± 0.37	4.23	4.20
**Basophils (Abs, ×10⁹/L)**	0.03 ± 0.11	0.04 ± 0.05	0.01	0.02
**Eosinophils (Abs, ×10⁹/L)**	0.19 ± 0.20	0.27 ± 0.33	0.16	0.20
**Lymphocytes (Abs, ×10⁹/L)**	2.11 ± 1.96	2.12 ± 0.97	1.95	2.00
**Platelets (×10⁹/L)**	251.32 ± 73.44	254.48 ± 73.23	244.00	246.00
**WBC (×10⁹/L)**	7.01 ± 3.24	7.31 ± 2.54	6.60	6.96

**Hemoglobin (g/dL)**	13.28 ± 1.48	13.53 ± 1.55	13.30	13.50
**IgA (mg/dL)**	236.83 ± 168.54	270.84 ± 139.93	212.00	245.00

5. <u>Definition of groups in Wiser</u>

5.1 For Cox group

- *Include all patients that:*

Have ANY of the following conditions:

IMMUNOGLOBULINS-E

Have ALL of the following conditions:

- Age [40,80]

Do NOT have ANY of the following conditions:

- RITUXIMAB [-1095,365]
- OMALIZUMAB [-545,365]
- DUPILUMAB [-545,365]
- METHYLPREDNISOLONE [-14,0] OR PREDNISOLONE [-14,0]
- 042* AIDS, ACQUIRED IMMUNODEFICIENCY VIRUS
- Date of Alia (Born in another country)
- Patient left clalit for good

5.2 For Kaplan-Meier groups

- *Include all patients that:*

Have ANY of the following conditions:

IMMUNOGLOBULINS-E IgE ≤ 25 IU/mL **or** IgE > 25 IU/mL

Have ALL of the following conditions:

- Age [40,80]

Do NOT have ANY of the following conditions:

- RITUXIMAB [-1095,365]
- OMALIZUMAB [-545,365]
- DUPILUMAB [-545,365]
- METHYLPREDNISOLONE [-14,0] OR PREDNISOLONE [-14,0]
- 042* AIDS, ACQUIRED IMMUNODEFICIENCY VIRUS

- Date of Alia (Born in another country)
- Patient left clalit for good

6. **ICD-9 codes used**

**Atopic dermatitis**	**691**
**Asthma**	493
**Allergic rhinitis**	477
**Smoking**	305.1
**Obesity**	278
**Urticaria**	708
**Alcoholic liver cirrhosis**	571.2

7. **Risk of groups less and more than 25 for male and female**

**Sex**	**IgE > 25 IU/mL – Risk (95% CI)**	**IgE ≤ 25 IU/mL – Risk (95% CI)**
**Male**	0.004149712 (0.003223673 – 0.00507489)	0.009531484 (0.006977245 – 0.012079154)
**Female**	0.002637584 (0.002037728 – 0.003237079)	0.004769547 (0.003667895 – 0.005869982)

8. **Relative Risk of Outcome According to IgE Level (≤25 IU/mL vs >25 IU/mL), by Sex**

**Sex**	**Relative Risk (RR)**	**95% Confidence Interval**
**Male**	**2.30**	**1.78 – 2.96**
**Female**	**1.81**	**1.39 – 2.34**

Relative risk (RR) was calculated by dividing the 7-year risk in participants with IgE ≤25 IU/mL by the risk in those with IgE > 25 IU/mL for each sex. The 95% confidence interval (CI) was derived using the log-transformed standard error of the risk ratio. Both males and females with low IgE exhibited a higher risk, with the association being stronger in males (RR = 2.30, 95% CI 1.78–2.96).

9. Assessment of Indication and Detection Bias

Because this was a retrospective cohort study, we systematically evaluated potential indication and detection bias associated with IgE testing. Diagnoses temporally associated with IgE measurement were grouped according to their typical clinical rationale. Conditions classically prompting IgE testing—such as allergic urticaria, angioedema, asthma, and atopic dermatitis—demonstrated markedly elevated relative risks, consistent with expected indication bias.

In contrast, autoimmune, hematologic, and systemic inflammatory diseases—where IgE testing is not routine—showed only modest risk elevations, while cardiometabolic and degenerative conditions exhibited minimal associations. This gradient of association supports the specificity of IgE-related findings and argues against generalized healthcare utilization as the sole explanation for the observed association between low IgE levels (<25 IU/mL) and subsequent CLL diagnosis.

Diagnostic group	Clinical rationale for IgE testing	Representative diagnoses*	Relative risk pattern†	Interpretation for bias
**A. Classic IgE-mediated allergic disease**	IgE measured as part of routine allergy evaluation	Allergic urticaria, idiopathic urticaria, angioedema, anaphylaxis, allergic rhinitis, asthma, food-related dermatitis	**Very high** (often >20; some >100)	**Strong indication bias**; expected and non-informative for CLL
**B. Chronic pruritic and eczematous skin disease**	IgE ordered in chronic inflammatory skin disorders	Atopic dermatitis, prurigo, chronic pruritus, contact dermatitis, dermatographism	**High** (≈10–150)	Reflects dermatologic work-up rather than causal association
**C. Eosinophilic and parasitic-related conditions**	IgE part of eosinophilia/parasitic evaluation	Eosinophilia, pruritic dermatoses, acariasis, scabies	**High** (≈10–260)	Confirms testing indication rather than

				disease prediction
**D. Chronic airway and sinonasal inflammation**	IgE measured in asthma–rhinitis overlap and chronic sinus disease	Chronic sinusitis, nasal polyps, chronic rhinitis, bronchiectasis	**Moderate** (≈5–70)	Allergy-driven evaluation; partial overlap with immune dysregulation
**E. Drug reactions and hypersensitivity syndromes**	IgE measured to assess suspected drug allergy	Drug-induced dermatitis, adverse drug reactions, unspecified allergy	**Moderate–high** (≈5–200)	Reflects clinical suspicion rather than disease causation
**F. Autoimmune and systemic inflammatory disease**	IgE not routinely indicated	SLE, rheumatoid arthritis, sarcoidosis, vasculitis	**Low–moderate** (≈3–30)	Suggests nonspecific immune activation, not indication bias
**G. Hematologic and lymphoproliferative conditions**	IgE not standard in diagnostic work-up	Mycosis fungoides, lymphadenopathy, plasma cell disorders	**Low–moderate** (≈3–15)	Possible immune dysregulation or diagnostic cascade
**H. Nonspecific symptoms and healthcare utilization markers**	Reflects increased clinical contact	Rash NOS, fatigue, abnormal labs, diagnostic procedures	**Low** (≈2–10)	**Detection bias** / surveillance effect
**I. Cardiometabolic and degenerative disease (negative control)**	No biologic link to IgE	Hypertension, diabetes, osteoarthritis, cataract	**Minimal** (≈1–4)	Supports specificity of IgE–CLL association

*Representative diagnoses derived from ICD codes listed in the supplemental dataset

†Relative risk patterns reflect observed sex-specific RRs, summarized by clinical group rather than individual codes.

PART B: sub-analysis

10. complete case analysis

Multivariable Cox Proportional Hazards Models for CLL – only patient with IgE and other immunoglobulin (IgG, IgA, IgM up to 3 months around the IgE). Patients aged between 40 and 80 years old (exclusion if received drugs, are HIV positive, quit CHS or born in another country): 20,338 patients. 68 patients with CLL.

Feature	HR (95% CI)
**Age**	**1.07 (1.05–1.10)**
**Male sex**	**1.93 (1.26–2.95)**
**Asthma**	0.67 (0.32–1.40)
**Allergic rhinitis**	1.17 (0.70–1.93)
**Smoking**	0.73 (0.38–1.38)
**Obesity**	1.44 (0.85–2.42)
**Urticaria**	0.44 (0.11–1.81)
**IgM deficiency**	0.97 (0.48–1.97)
**IgA deficiency**	2.12 (0.73–6.19)
**Low IgE (<25 IU/mL)**	**1.94 (1.27–2.97)**
**Hypogammaglobulinemia (adult)**	**2.20 (0.97–5.01)**

As seen, the lower level of IgE still be statistically significant.

11. piecewise Cox proportional hazards

Multivariable Cox Proportional Hazards Models for CLL (by follow-up period)

Feature	0–2 years HR (95% CI)	2–4 years HR (95% CI)	4–7 years HR (95% CI)
**Age**	**1.07 (1.05–1.10)**	**1.08 (1.05–1.11)**	**1.04 (1.02–1.07)**
**Male sex**	**1.67 (1.07–2.61)**	**1.88 (1.11–3.19)**	**1.85 (1.14–3.01)**
**Atopic dermatitis**	1.30 (0.32–5.31)	NE	NE
**Asthma**	0.47 (0.20–1.10)	0.78 (0.35–1.74)	1.53 (0.84–2.79)
**Allergic rhinitis**	0.93 (0.52–1.66)	1.43 (0.78–2.63)	1.18 (0.66–2.12)
**Smoking**	1.05 (0.57–1.92)	0.85 (0.38–1.89)	1.35 (0.71–2.53)
**Obesity**	1.22 (0.70–2.15)	1.10 (0.54–2.24)	0.60 (0.26–1.39)
**Urticaria**	0.51 (0.12–2.07)	1.53 (0.55–4.24)	0.91 (0.29–2.91)
**IgM deficiency**	1.95 (0.78–4.88)	0.71 (0.09–5.43)	1.68 (0.40–7.06)
**IgA deficiency**	2.09 (0.56–7.75)	5.61 (0.74–42.65)	NE
**Hypogammaglobulinemia**	**7.97 (3.37–18.83)**	NE	1.80 (0.24–13.45)
**Low IgE (<25 IU/mL)**	**2.13 (1.36–3.31)**	**1.94 (1.14–3.28)**	**1.75 (1.07–2.86)**
			NE: not estimable

As seen, the lower level of IgE still be statistically significant over time.

12. cox regression with IgE less than 25 IU/mL (7 years) using data from 2007 to 2023

Feature	0–7 Years	2–7 Years
**Male sex**	**1.84 (1.37–2.46)**	**1.89 (1.30–2.75)**
**Age (per year)**	**1.07 (1.05–1.08)**	**1.06 (1.04–1.08)**
**Atopic dermatitis**	0.58 (0.14–2.32)	NE: not estimable
**Asthma**	0.86 (0.56–1.32)	1.12 (0.67–1.85)
**Allergic rhinitis**	1.16 (0.82–1.64)	1.34 (0.87–2.07)
**Tobacco use disorder**	1.09 (0.74–1.62)	1.10 (0.66–1.83)
**Obesity**	1.07 (0.27–4.32)	1.70 (0.42–6.89)
**Urticaria**	0.88 (0.43–1.78)	1.12 (0.49–2.55)
**Low IgE (≤25 IU/mL)**	**1.82 (1.36–2.43)**	**1.76 (1.21–2.57)**
**IgM deficiency (<40 mg/dL)**	1.68 (0.82–3.44)	1.32 (0.40–4.28)
**IgA deficiency (adult)**	2.62 (0.88–7.84)	2.42 (0.31–18.79)
**Hypogammaglobulinemia (adult)**	**4.31 (2.03–9.17)**	0.83 (0.11–6.37)
		NE: not estimable

13. Cox regression for 7 years (per laboratory)

Multivariable Cox Proportional Hazards Models for CLL – every laboratory with IgE of patients between 40 and 80 years old (exclusion if received drugs, are HIV positive, quit CHS or born in another country). Possible several laboratories for the same patient: 176,337 laboratories.

**Feature**	**0–7 years HR (95% CI)**	**2–7 years HR (95% CI)**
**Male sex**	**1.84 (1.37–2.46)**	**1.89 (1.30–2.75)**
**Age (per year)**	**1.07 (1.05–1.08)**	**1.06 (1.04–1.08)**
**Atopic dermatitis**	0.58 (0.14–2.32)	NE (not estimable)
**Asthma**	0.86 (0.56–1.32)	1.12 (0.67–1.85)
**Allergic rhinitis**	1.16 (0.82–1.64)	1.34 (0.87–2.07)
**Tobacco use disorder**	1.09 (0.74–1.62)	1.10 (0.66–1.83)
**Obesity**	1.07 (0.27–4.32)	1.70 (0.42–6.89)
**Urticaria**	0.88 (0.43–1.78)	1.12 (0.49–2.55)
**Low IgE (≤25 IU/mL)**	1.82 (1.36–2.43)	1.76 (1.21–2.57)
**IgM deficiency (<40 mg/dL)**	1.68 (0.82–3.44)	1.32 (0.40–4.28)
**IgA deficiency (adult)**	2.62 (0.88–7.84)	2.42 (0.31–18.79)
**Hypogammaglobulinemia (adult)**	**4.31 (2.03–9.17)**	**0.83 (0.11–6.37)**

14. Considering lymphocyte

14.1 Cox regression for 7 years considering Lymphocyte as continuous variable

Feature	Hazard Ratio (95% CI)
**Male sex**	**1.38 (0.97–1.96)**
**Age (per year)**	**1.07 (1.05–1.09)**
**Atopic dermatitis**	0.43 (0.06–3.10)
**Asthma**	0.88 (0.54–1.44)
**Allergic rhinitis**	1.38 (0.92–2.07)
**Tobacco use disorder**	1.19 (0.75–1.90)
**Obesity**	0.83 (0.12–5.91)
**Urticaria**	0.80 (0.33–1.96)
**Low IgE (≤25 IU/mL)**	**1.58 (1.11–2.25)**
**IgM deficiency**	0.77 (0.21–2.86)
**IgA deficiency**	1.77 (0.42–7.56)
**Hypogammaglobulinemia (adult)**	0.69 (0.14–3.28)
**Absolute lymphocyte count ($\times 10^9$/L)**	**1.08 (1.07–1.09)**

14.2 Cox regression for 7 years Lymphocyte as categorial (less than 4.8 ($\times 10^9$/L))

Feature	Hazard Ratio (95% CI)
**Male sex**	**1.81 (1.38–2.37)**
**Age (per year)**	**1.07 (1.05–1.08)**
**Atopic dermatitis**	0.46 (0.11–1.83)
**Asthma**	0.87 (0.58–1.31)
**Allergic rhinitis**	1.10 (0.79–1.54)
**Tobacco use disorder**	1.11 (0.76–1.62)
**Obesity**	1.01 (0.25–4.06)
**Urticaria**	1.04 (0.57–1.91)
**Low IgE (≤25 IU/mL)**	**2.02 (1.54–2.65)**
**IgM deficiency**	1.56 (0.79–3.10)
**IgA deficiency**	2.70 (0.99–7.34)
**Hypogammaglobulinemia (adult)**	**3.40 (1.59–7.27)**
**Lymphocytes <4.8×10^9/**	0.73 (0.18–2.95)

15. atopic and non-atopic Kaplan-Meier curve

Atopy was defined as apparition any time before the diagnosis of Atopic Dermatitis (ICD-9 : 691), or Asthma (ICD-9 : 493) or allergic rhinitis (ICD-9 : 477)

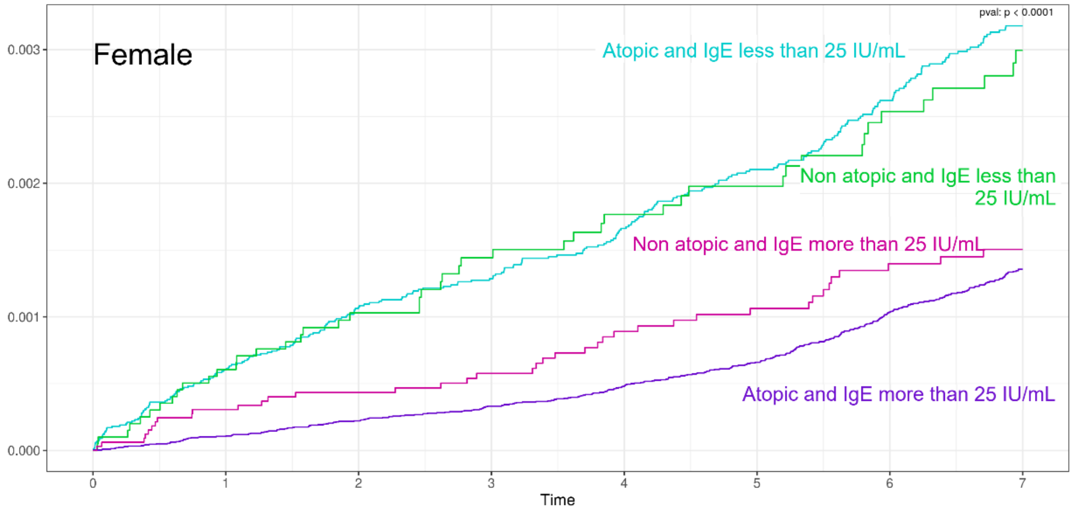

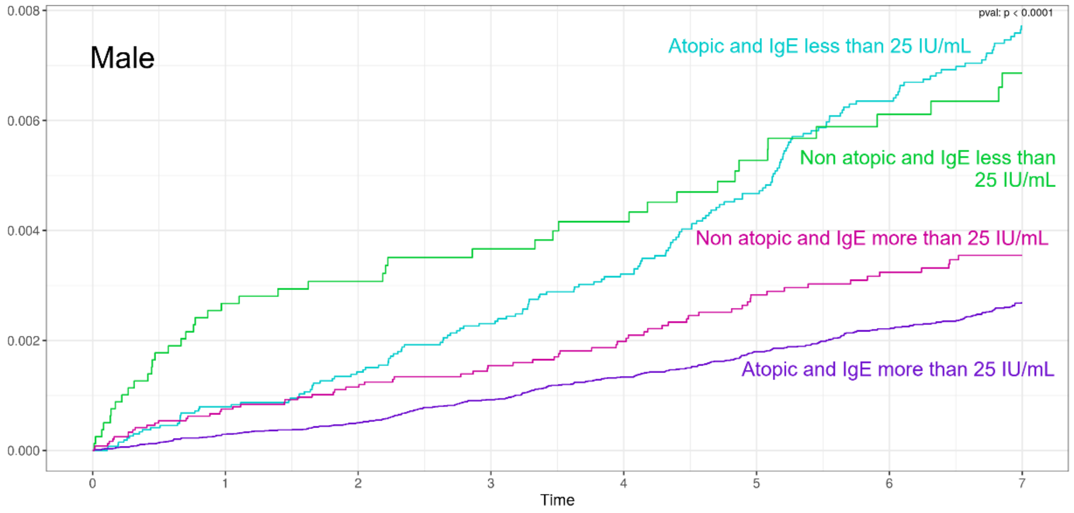

As seen, the lower level of IgE is more associated with higher risk of CLL than the atopic status.

16. Kaplan-Meir curve and Cox regression for 10 years

16.1 Kaplan-Meir for 10 years

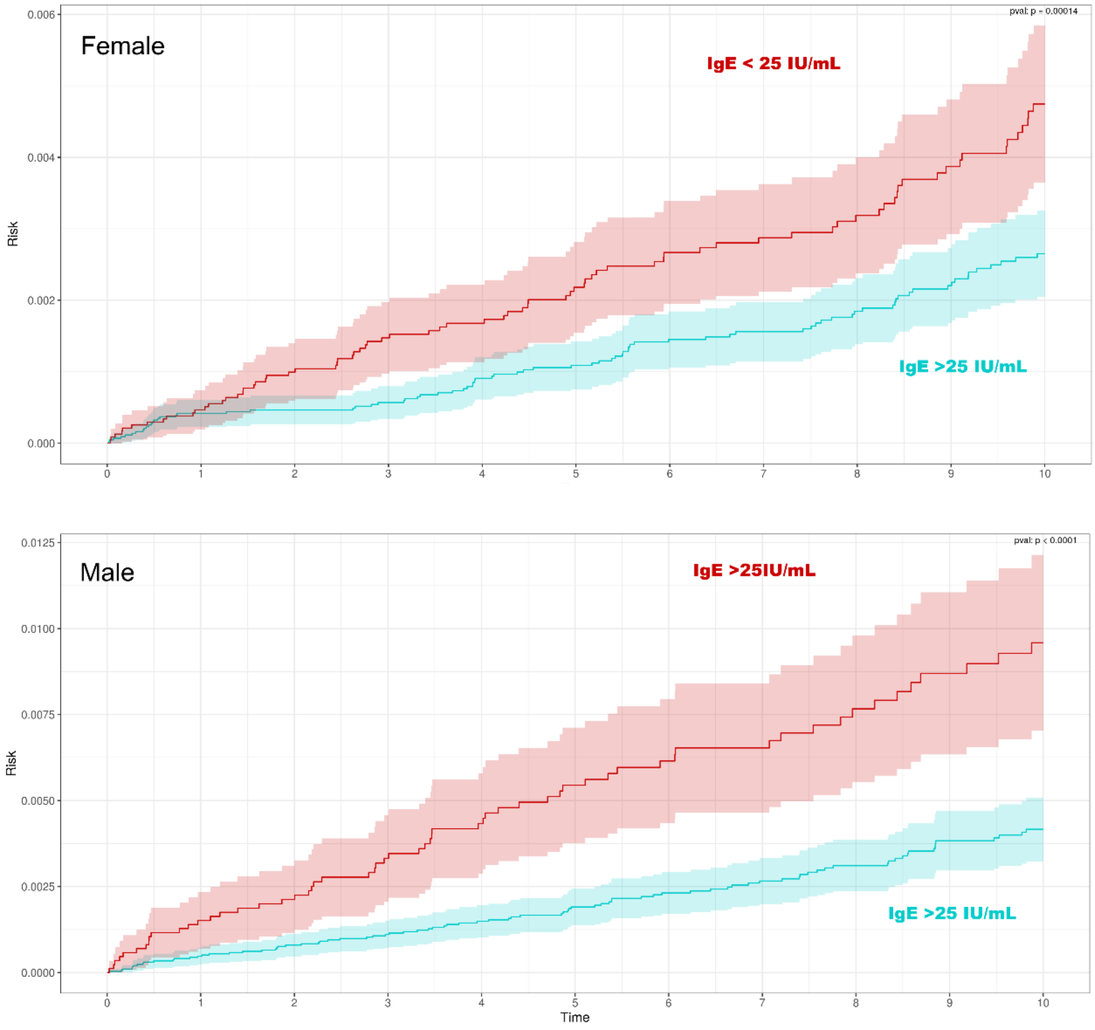

16.2 Cox regression for 10 years

**Feature**	**0–10 yearsHR (97.5% CI)**	**2–10 yearsHR (97.5% CI)**	**7–10 yearsHR (97.5% CI)**
**Age**	**1.06 (1.05–1.07)**	**1.05 (1.04–1.07)**	**1.04 (1.02–1.06)**
**Sex, male**	**1.66 (1.31–2.09)**	**1.65 (1.25–2.17)**	1.27 (0.80–2.01)
**Atopic dermatitis**	0.67 (0.25–1.81)	0.47 (0.12–1.89)	1.33 (0.33–5.40)
**Asthma**	0.85 (0.59–1.20)	1.00 (0.68–1.48)	0.74 (0.35–1.56)
**Allergic rhinitis**	0.98 (0.72–1.32)	1.03 (0.72–1.46)	0.68 (0.34–1.36)
**Smoking**	1.30 (0.94–1.78)	1.36 (0.94–1.98)	**1.84 (1.02–3.32)**
**Obesity**	0.77 (0.19–3.11)	1.03 (0.26–4.15)	Not estimable
**Urticaria**	0.80 (0.45–1.43)	0.84 (0.43–1.63)	0.23 (0.03–1.67)
**Low IgE (≤ 25 IU/mL)**	**1.93 (1.53–2.44)**	**1.90 (1.44–2.50)**	**1.68 (1.06–2.65)**
**IgM deficiency**	1.52 (0.82–2.83)	1.32 (0.57–3.08)	1.26 (0.29–5.38)
**IgA deficiency**	**3.56 (1.52–8.36)**	**5.26 (1.85–14.98)**	**7.87 (1.84–33.77)**
**Hypogammaglobulinemia**	**2.39 (1.13–5.08)**	0.31 (0.04–2.37)	Not estimable

PART C: Kaplan-Meir and COX regression with threshold from the literature

17.1 Kaplan-Meier curves for IgE less and more than 20 IU/mL

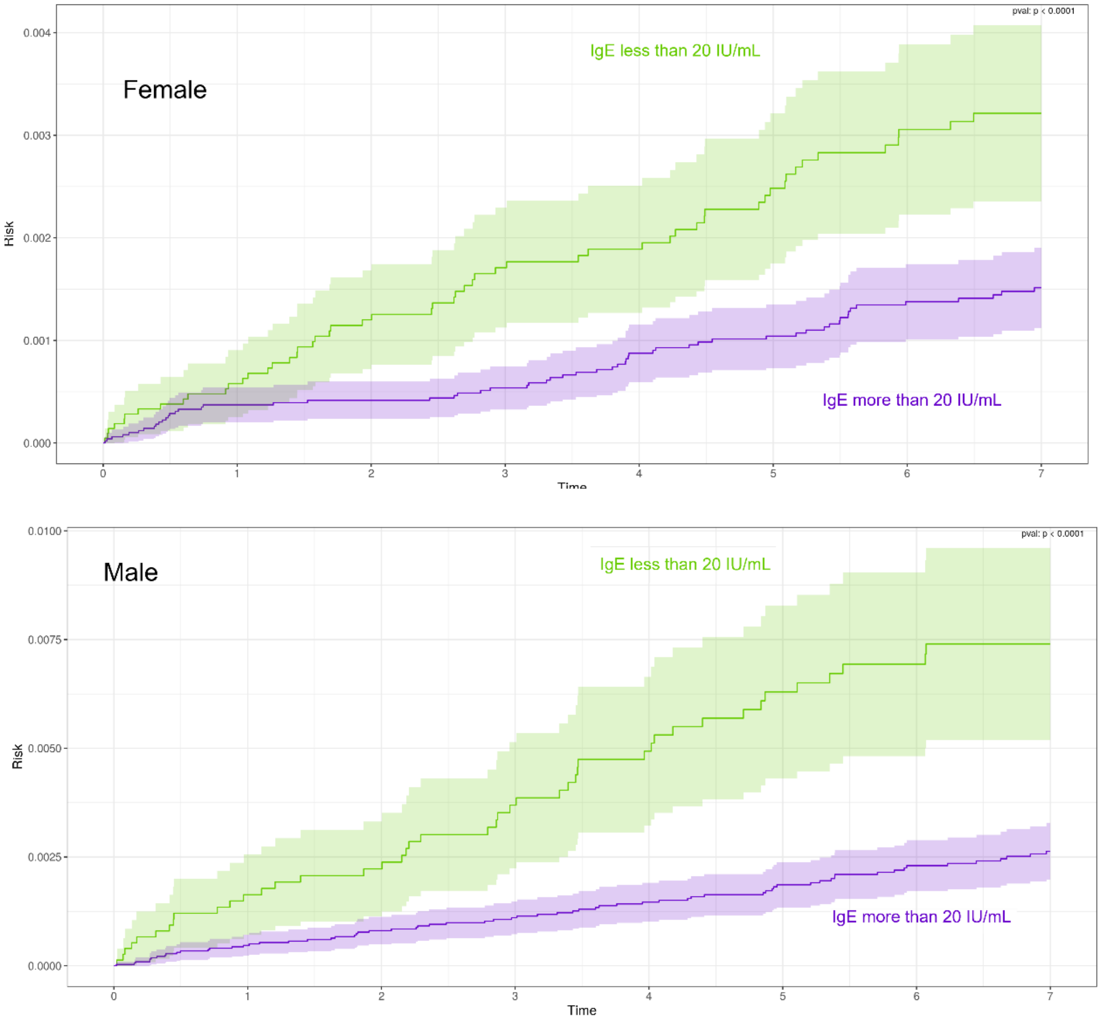

17.2 Cox regression with IgE less than 20 IU/mL (7 years)

**Feature**	**HR (95% CI)**
**Age**	**1.07 (1.05–1.08)**
**Male sex**	**1.84 (1.39–2.43)**
**Atopic dermatitis**	0.50 (0.12–2.03)
**Asthma**	0.88 (0.59–1.34)
**Allergic rhinitis**	1.13 (0.80–1.59)
**Smoking**	1.11 (0.76–1.63)
**Obesity**	1.01 (0.68–1.49)
**Urticaria**	0.91 (0.47–1.78)
**IgM deficiency**	1.57 (0.77–3.22)
**IgA deficiency**	2.15 (0.72–6.44)
**Hypogammaglobulinemia**	**3.98 (1.88–8.42)**
**Low IgE (<20 IU/mL)**	**2.19 (1.65–2.89)**
